# Tumor Necrosis Factor, but Not Neutrophils, Alters the Metabolic Profile in Acute Experimental Arthritis

**DOI:** 10.1371/journal.pone.0146403

**Published:** 2016-01-07

**Authors:** Marina C. Oliveira, Luciana P. Tavares, Juliana P. Vago, Nathália V. Batista, Celso M. Queiroz-Junior, Angelica T. Vieira, Gustavo B. Menezes, Lirlândia P. Sousa, Fons A. J. van de Loo, Mauro M. Teixeira, Flávio A. Amaral, Adaliene V. M. Ferreira

**Affiliations:** 1 Department of Nutrition, Nursing School, Universidade Federal de Minas Gerais, Belo Horizonte, Minas Gerais, Brazil; 2 Immunopharmacology, Department of Biochemistry and Immunology, Institute of Biological Sciences, Universidade Federal de Minas Gerais, Belo Horizonte, Minas Gerais, Brazil; 3 Department of Clinical and Toxicological Analyses, Faculty of Pharmacy, Universidade Federal de Minas Gerais, Belo Horizonte, Minas Gerais, Brazil; 4 Department of Clinic, Pathology and Odontological Surgery, Faculty of Odontology, Universidade Federal de Minas Gerais, Belo Horizonte, Minas Gerais, Brazil; 5 Department of Morphology, Institute of Biological Sciences, Universidade Federal de Minas Gerais, Belo Horizonte, Minas Gerais, Brazil; 6 Experimental Rheumatology, Radboud university medical center, Nijmegen, The Netherlands; University of Sao Paulo, BRAZIL

## Abstract

Metabolic alterations are associated with arthritis apart from obesity. However, it is still unclear which is the underlying process behind these metabolic changes. Here, we investigate the role of tumor necrosis factor (TNF) in this process in an acute model of antigen-induced arthritis (AIA). Immunized male BALB/c mice received an intra-articular injection of PBS (control) or methylated bovine serum albumin (mBSA) into their knees, and were also pre-treated with different drugs: Etanercept, an anti-TNF drug, DF2156A, a CXCR1/2 receptor antagonist, or a monoclonal antibody RB6-8C5 to deplete neutrophils. Local challenge with mBSA evoked an acute neutrophil influx into the knee joint, and enhanced the joint nociception, along with a transient systemic metabolic alteration (higher levels of glucose and lipids, and altered adipocytokines). Pre-treatment with the conventional biological Etanercept, an inhibitor of TNF action, ameliorated the nociception and the acute joint inflammation dominated by neutrophils, and markedly improved many of the altered systemic metabolites (glucose and lipids), adipocytokines and PTX3. However, the lessening of metabolic changes was not due to diminished accumulation of neutrophils in the joint by Etanercept. Reduction of neutrophil recruitment by pre-treating AIA mice with DF2156A, or even the depletion of these cells by using RB6-8C5 reduced all of the inflammatory parameters and hypernociception developed after AIA challenge, but could not prevent the metabolic changes. Therefore, the induction of joint inflammation provoked acute metabolic alterations which were involved with TNF. We suggest that the role of TNF in arthritis-associated metabolic changes is not due to local neutrophils, which are the major cells present in this model, but rather due to cytokines.

## Introduction

Systemic metabolic alterations are not only caused by obesity and their associated comorbidities, but also linked to autoimmune diseases such as arthritis [1]. Arthritis is characterized by an infiltration of inflammatory cells, cartilage and bone destruction, and it is clinically presented as pain, swelling and stiffness of affected joints [2]. Inflammatory cytokines and chemokines play a pivotal role in the local and systemic inflammation of arthritic patients, contributing to the disease development and progression [3]. Despite it is not well explored, neutrophils also participate in arthritis progression, and evidences indicate that neutrophil influx does occur during recurrence of disease [4]. Moreover, lean patients diagnosed with arthritis have shown alterations in serum levels of adipocytokines, which are released mainly from the adipose tissue and are also associated with arthritis progression [5].

Different categories of drugs are routinely used for the treatment of arthritis, aiming to relieve symptoms and avoid progression of the disease [6]. Although some components of the arthritic inflammatory response still need to be unveiled, there have been significant developments in the last decades, including novel immunobiological agents targeting tumor necrosis factor (TNF). In addition to providing relief to patients, these agents have been shown to improve metabolic alterations associated with arthritis [7, 8]. However, mechanisms describing the relationship among soluble mediators and systemic metabolic alterations still need to be elucidated.

We have previously described a local production of TNF-α and chemokine (C-X-C motif) receptor 2 (CXCR2)-mediated neutrophil influx following antigen challenge in a model of antigen-induced arthritis (AIA) in mice [9, 10]. Here, we report that there is also a systemic metabolic alteration after acute induction of AIA. We then investigated the relative contribution of TNF for the observed systemic metabolic changes, and which is the relation to its known inflammatory role.

## Materials and Methods

### Ethical Approval

All experiments with mice were approved by the “Ethics Committee in Animal Experimentation at Universidade Federal de Minas Gerais” in Brazil (protocol: 148/2012).

### Animals

Eight-week-old male BALB/c mice were obtained from the animal care center at Universidade Federal de Minas Gerais. It was also used mice with a lysozyme M promoter for enhanced green fluorescent protein (LysM-eGFP), expressing fluorescently neutrophils for the confocal microscopy analysis. They were maintained in an environmentally controlled room under a 12/12 h light-dark cycle, with filtered water and food *ad libitum*. During the procedures for arthritis induction, mice were anesthetized with 1.5% isoflurane in oxygen. After the indicated time points, mice were anesthetized with ketamine (80 mg/kg) and xylazine (10 mg/kg) and killed. Samples of the blood, knee, epididymal adipose tissue and liver were collected for further analysis.

### Arthritis induction and assessment of articular inflammation

Mice were immunized i.d. at the base of the tail with 500 μg of methylated BSA (mBSA) in 100 μL of an emulsion containing saline and an equal volume of complete Freund’s adjuvant. The knee challenge was performed 14 days later. For the control group, each mouse received an intra-articular injection in both knee joints with 10 μL PBS. This group is represented by the mean of all time points evaluated. For AIA mice, they were injected with 10 μg mBSA in 10 μL PBS [9]. After the antigen challenge, mice were killed at the indicated time points. The knee cavity was washed with PBS (2x 5 μL) for cell recovery. The total number of leukocytes was determined by counting leukocytes in the synovial fluid by manual counting using Neubauer chamber under optical microscopy after staining with Turk’s solution. Differential counts were obtained from cytospin preparations (Shandon III, Thermo Shandon, Frankfurt, Germany) stained with May—Grünwald—Giemsa. The peri-articular tissue was removed for determination of myeloperoxidase activity and cytokine/chemokine measurement by ELISA.

### Drugs

Etanercept (Enbrel^®^) was diluted in saline and administered i.p. (10 mg/kg) in a volume of 100 μL 30 min before the arthritis induction. The allosteric CXCR1/2 inhibitor DF2156A (10 mg/kg) was diluted in carboxymethylcellulose 0.5% and given orally per gavage 30 min before arthritis challenge (Biogen-Dompé, Italy). Anti-GR-1 MAb (RB6-8C5 clone) was injected i.v. (1 mg/mL) in a volume of 100 μL twice, one day and 1 hour before arthritis induction (eBioscience, San Diego, CA). Control PBS and AIA groups received the same vehicle and administration route of the drug correspondent in each experiment.

### Nociception assessment

In a quiet room, the mice were placed in acrylic cages (12x10x17 cm high) with a wire grid floor, 15–30 min before the test, for environmental adaptation. A series of stimuli was performed only when the animals were quiet, without exploratory movements or defecation. In these experiments, an electronic pressure meter was used. It consists of a hand-held force transducer fitted with a polypropylene tip (INSIGHT Instruments, Ribeirão Preto, São Paulo, Brazil) [11]. A non-standard large tip (4.15 mm^2^) was adapted to the probe [12]. An increasing perpendicular force was applied to the central area of the plantar surface of the hind paw to induce the flexion of the knee joint, followed by paw withdrawal. A tilted mirror below the grid provided a clear view of the animal’s hind paw. The end point was characterized by the removal of the paw from the polypropylene tip. After the flexion-elicited withdrawal threshold, the intensity of the pressure was automatically recorded. The value for the response was obtained by averaging two measurements in one hind paw.

### Total and differential blood cell counts

Blood was collected from the tail vein of mice and the total white blood cells were counted using a Neubauer chamber. Peripheral blood smears were stained with May-Grünwald-Giemsa, and the differential white blood cell count was determined under oil immersion (1000x).

### Histology

The knee joint was removed and fixed for 24 h with 4% a neutral-buffered formalin. Then, the joints were incubated in 14% EDTA at pH 7.2 during 4 weeks at room temperature for decalcification. The samples were embedded in paraffin and sections of 7 mm were stained with Hematoxilin-eosin. Two sections/knee joint were microscopically examined and scored in a blind manner for different parameters [13], as follows: severity of synovial hyperplasia (ranging from 0 to 3) and intensity and extension of inflammatory infiltrate (ranging from 0 to 4). The grades were summed to obtain an arthritis index (ranging from 0 to 7).

### Confocal Microscopy

Immunized LysM-eGFP mice (eGFP-expressing neutrophils) were challenge with a knee injection of PBS or mBSA and after 3 and 24 hours the confocal microscopy was performed as described before [14]. Briefly, mice were anesthetized with ketamine (80 mg/kg) and xylazine (10 mg/kg). Epididymal adipose tissue and liver were exposed over a Plexiglas support, and the tissue microcirculation was visualized under confocal microscopy using an Olympus Fluoview FV300 laser-scanning microscope equipped with a 488 nm argon laser. All images were acquired using a 10x magnification objective lens, and three different fields from each mouse were analyzed.

### Oral glucose tolerance test (OGTT)

After 24 hours of PBS or mBSA knee injection, mice were fasted overnight and received D-glucose (2 mg/g body weight) orally per gavage. Levels of glucose were measured from the blood collected from the tip of the tail at 0, 15, 30, 60, and 90 min after D-glucose administration using a glucometer (Accu-Check, Roche Diagnostics, Indianapolis, IN).

### Metabolic analyses in the serum

Total cholesterol, triglyceride and glucose levels were quantified by enzymatic kits (KATAL, Belo Horizonte, MG, Brazil). Adiponectin, resistin and leptin levels were assayed by ELISA (R&D systems Europe Ltd., Abington, UK) as well as insulin (Millipore, Bedford, MA). The insulin resistance index was calculated as follows: HOMA-IR = fasting glucose level (mmol/L) x fasting insulin level (μU/mL) ÷ 22.5.

### ELISA assay

Samples of peri-articular tissue, epididymal adipose tissue and liver were homogenized with a protease inhibitor solution (1:10). This solution contained 0.4 M NaCl, 0.05% Tween 20, 0.5% bovine serum albumin, 0.1 mM fluoride fenilmetilsufonila, 0.1 mM benzethonium chloride, 10 mM EDTA, 20 IU of aprotinin diluted in a solution of phosphate buffer (8g NaCl, 0.2g KCl and 2.89 g Na_2_HPO_4_.12H_2_O diluted in 1 liter of distilled water). The homogenate was centrifuged at 10,000 rpm for 10 minutes at 4°C and the supernatant of knee and liver, and the infranatant of adipose tissue were collected to measure cytokine/chemokine levels by ELISA. Levels of chemokine (C-X-C motif) ligand 1 (CXCL1) were determined in the peri-articular tissue, epididymal adipose tissue and liver, and pentraxin 3 (PTX3) was evaluated in the serum using DuoSet ELISA development kits (R&D System, Inc., Minneapolis, MN, USA), in which cytokines are detected using a sandwich antibody system according to the manufacturer’s instructions.

### Myeloperoxidase (MPO) activity

Indirect neutrophil presence was measured by assaying MPO activity, as described previously [15]. Briefly, peri-articular tissue, epididymal adipose tissue and liver samples of mice were homogenized and assayed for MPO activity by measuring the change in OD at 450 nm using tetramethylbenzidine.

### Leukocyte analysis by flow cytometry

After blood collection (0.1 ml), the red blood cells were lysed with ACK buffer. Subsequently, cells were incubated with the specific monoclonal antibody for markers of the main cellular populations present in the peripheral blood, GR1 and CD11b (neutrophils), CD3 (T cells) and F4/80 (monocytes) (BD Pharmingen). Cells were analyzed with a FACSCalibur CantoII, and data were analyzed by FlowJo (TreeStar).

### Statistical analysis

Data are expressed as mean ± SEM. Multiple comparisons were performed using one-way ANOVA: it was used the Dunnett’s post-hoc test in the kinetic results; for experiments with drugs the Newman-Keuls post-hoc analysis and; in the OGTT two-way ANOVA followed by the Bonferroni post-hoc test. The statistical analysis was performed using the software GraphPad Prism (GraphPad Software, La Jolla, CA). The statistical significance was set at *P*<0.05.

## Results

### Acute arthritis induces accumulation of neutrophils in the adipose tissue and liver, and triggers metabolic alterations

**Fig 1:**
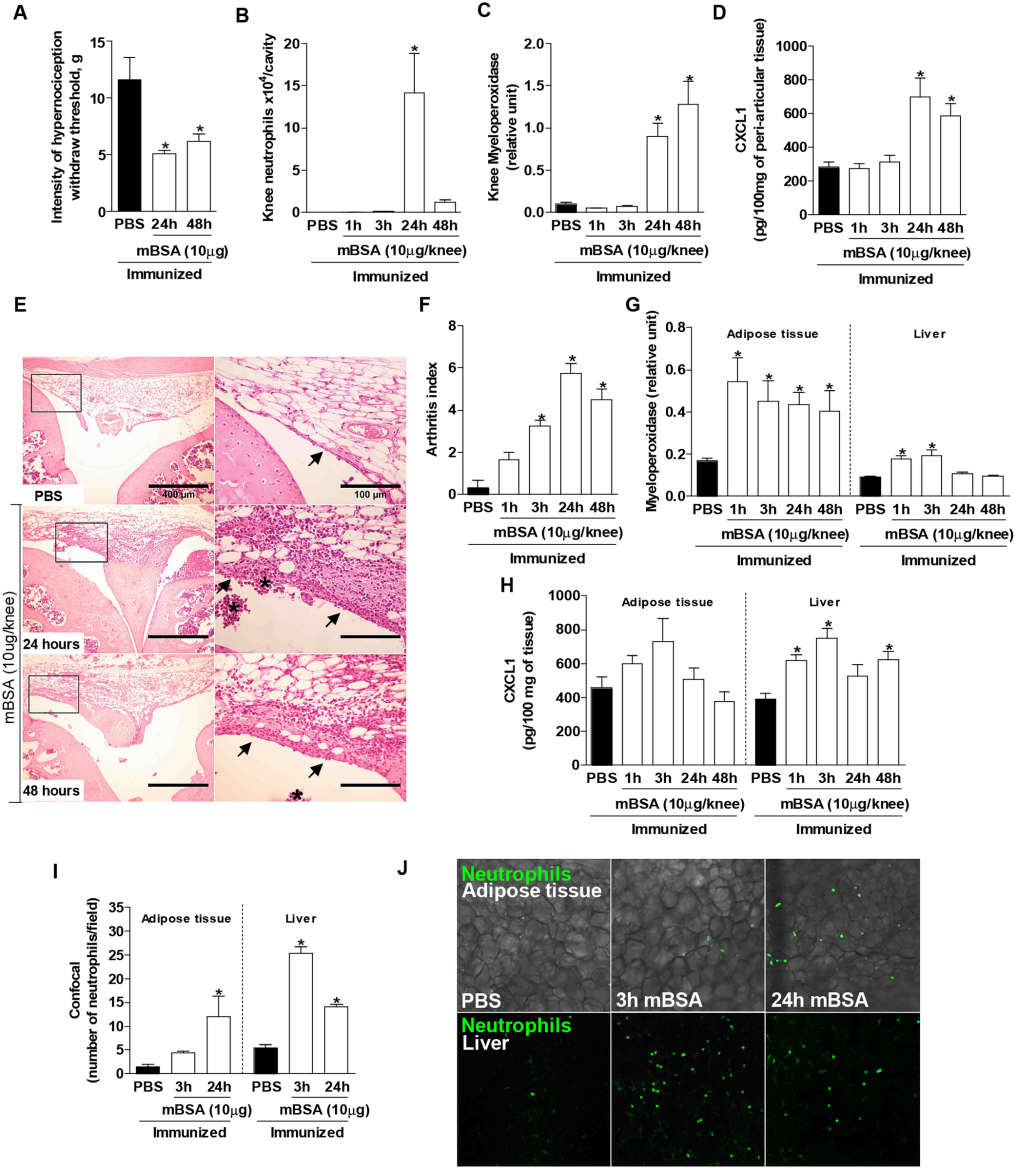
Antigen-induced arthritis in mice is mainly associated with the presence of neutrophils. (A) Intensity of nociception. (B) Number of neutrophils, (C) myeloperoxidase activity, and (D) CXCL1 chemokine levels in the peri-articular tissue. (E) Representative photos of the morphologic alterations in the knee (x100 and x400) and (F) arthritis index. Arrows indicate the synovial and asterisks represent the inflammatory infiltrate. Adipose tissue and liver (G) myeloperoxidase activity, and (H) CXCL1 levels at 1, 3, 24 and 48 hours after the challenge with the antigen-induced arthritis (AIA). Bars represent the mean values±SEM (n = 6–8). Neutrophils number in (I) epididymal adipose tissue and liver accumulated in Lysm-eGFP mice and (J) representative confocal microscopy image, 3 and 24 hours after the challenge with the AIA (x100). Bars represent the mean values±SEM (n = 3–5), *P<0.05 vs. PBS.

At 24 and 48 hours after AIA induction, mice had a significant hypernociception, which is an index of pain, as assessed by the lower paw withdrawal threshold compared with control mice (Fig 1A). This acute phase of AIA was also characterized by an increased recruitment of neutrophils into the knee joint and peri-articular tissue (MPO activity), which peaks at 24 hours after challenge (Fig 1B and 1C). Neutrophil accumulation in the synovial fluid resolved at 48 hours, but it was still present at this time in the peri-articular tissue. The recruitment of neutrophils was mirrored by the presence of the neutrophil-active chemokine CXCL1 in the peri-articular tissue (Fig 1D). Histopathological analysis showed an intense infiltration of neutrophils and increased synovial hyperplasia (Fig 1E and 1F). There was also an increase in MPO activity in the epididymal adipose tissue and liver, suggesting a significant influx of neutrophils in these two organs (Fig 1G). Despite this increase in neutrophil infiltration, we did not observe alterations in the levels of CXCL1 chemokine in the adipose tissue, but only in the liver at 1, 3 and 48 hours after AIA challenge (Fig 1H). Using Lysm-eGFP mice, which present eGFP-expressing neutrophils, we showed an increase in the neutrophil accumulation in the adipose tissue at 24 hours and in the liver at 3 and 24 hours following arthritis induction (Fig 1I and 1J), concurring with the MPO data.

**Fig 2:**
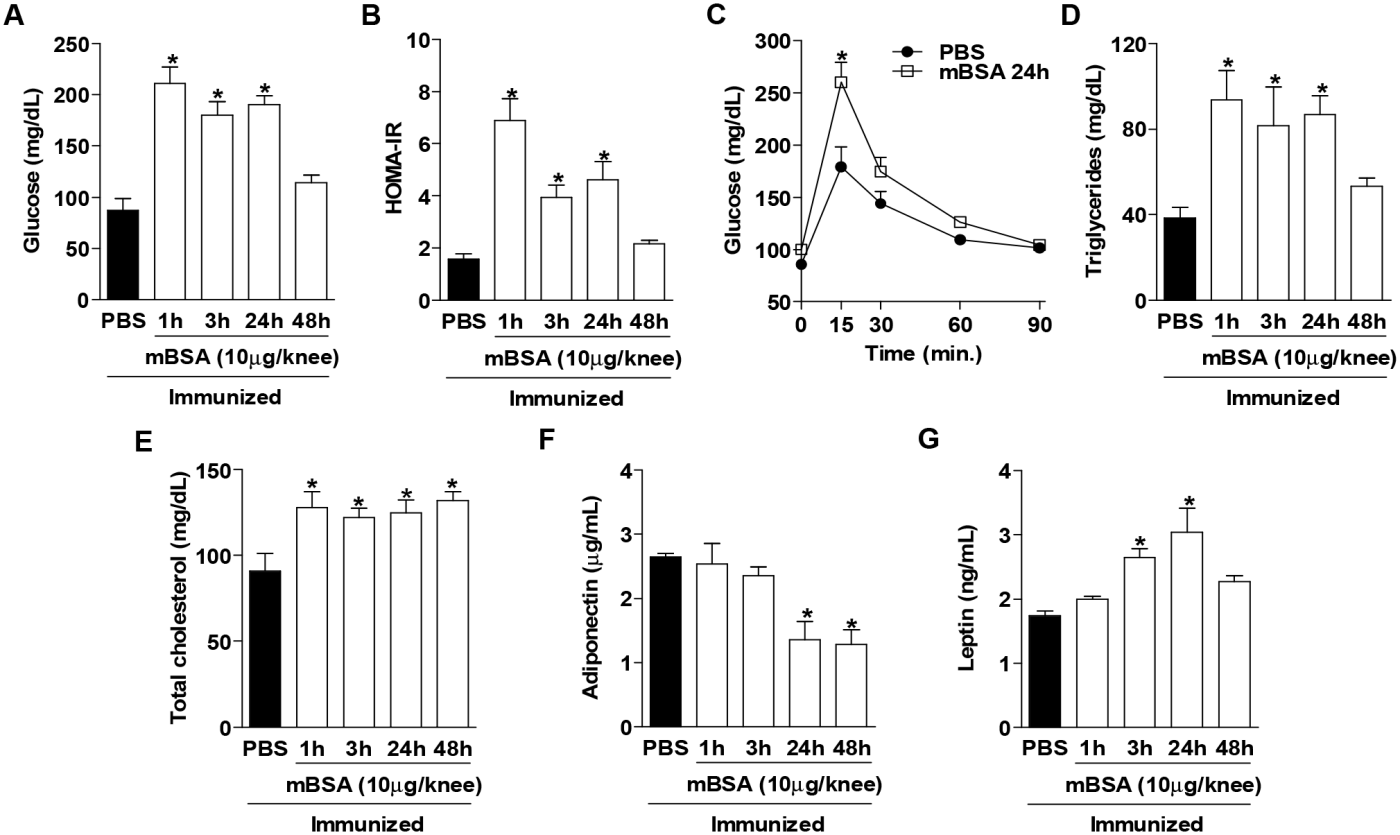
Systemic metabolic alterations in antigen-induced arthritis mice at different time points. The metabolism of glucose showed by (A) glucose levels, (B) HOMA-IR index and (C) oral glucose tolerance test. The lipid metabolism represented by (D) triglyceride and (E) total cholesterol levels. Serum levels of the adipocytokines, (F) adiponectin and (G) leptin at 1, 3, 24 and 48 hours after the challenge with the antigen-induced arthritis (AIA). The bars represent the mean values ± SEM (n = 6–8), *P<0.05 vs. PBS.

In order to verify whether the metabolism of mice was affected when subjected to AIA, we evaluated the major systemic metabolic parameters after antigen administration in the joint of immunized mice. AIA mice showed alterations in the glucose and lipid metabolism, as seen by an acute hyperglycemia, insulin resistance, glucose intolerance, hypertriglyceridemia and increased cholesterol levels (Fig 2A–2E). The adipocytokines, which are released mainly by adipose tissue, were also altered in AIA mice. Levels of adiponectin reduced while levels of leptin increased in specific time points (Fig 2F and 2G). There were no changes in resistin levels (data not shown). Altogether, these results demonstrate that mice challenged with a local antigen-specific inflammatory stimulus had significant systemic metabolic changes.

### The metabolic changes in AIA mice are dependent of TNF

**Fig 3:**
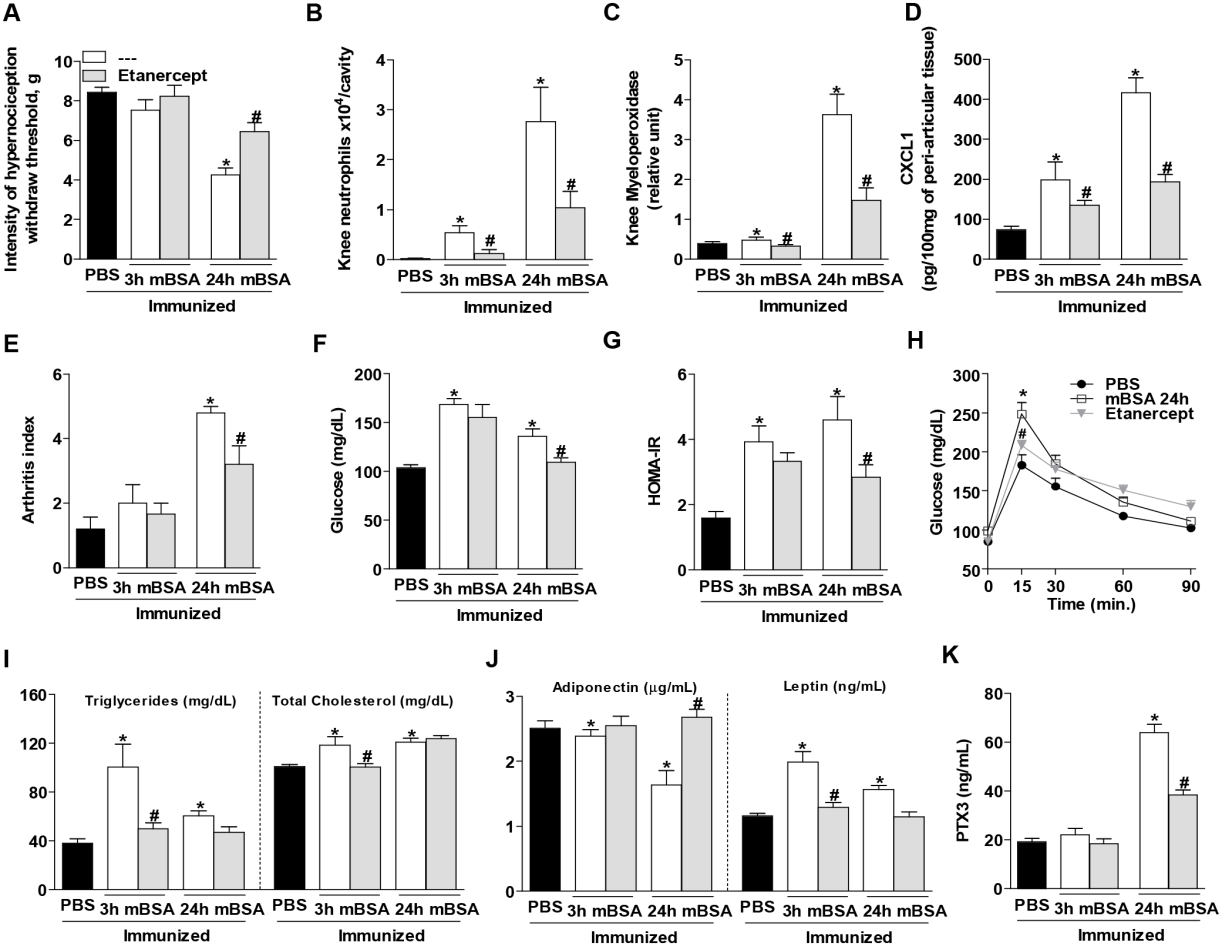
Pre-treatment with Etanercept improves the altered metabolic parameters of antigen-induced arthritis mice. (A) Intensity of nociception. (B) Neutrophils, (C) myeloperoxidase activity, (D) CXCL1 chemokine in the peri-articular tissue and (E) arthritis index. Systemic glucose metabolism showed by (F) glucose levels, (G) HOMA-IR index and (H) oral glucose tolerance test. The lipids, (I) triglyceride and total cholesterol levels. The adipocytokines, (J) adiponectin and leptin levels. (K) PTX3 levels of mice that received a knee intra-articular injection of PBS, mBSA or mBSA treated prior with Etanercept. Bars represent the mean values ±SEM (n = 6–8), *P<0.05 vs. PBS; #*P*<0.05 vs. AIA of the respective time.

The blockade of TNF may prevent the inflammatory response and hypernociception in this model of AIA [10]. To evaluate the relevance of TNF in the systemic metabolic changes observed after induction of AIA, mice were treated with Etanercept, a known TNF inhibitor in humans and mice [16, 17]. The pre-treatment with Etanercept reduced all the inflammatory parameters developed after AIA challenge (Fig 3A–3E). Interestingly, glucose and lipid parameters were also improved (Fig 3F–3I), and serum levels of adiponectin and leptin were normalized in mice treated with Etanercept (Fig 3J). PTX3 is a serum reactive protein rapidly produced during inflammatory responses [18]. Interestingly, Etanercept also prevented the increase in PTX3 levels observed after induction of AIA (Fig 3K). Therefore, TNF appears to represent an important component for the altered systemic metabolic parameters in this model of AIA.

### Although neutrophils are infiltrated into the adipose tissue and liver, they do not appear to alter metabolic parameters induced by AIA

**Fig 4:**
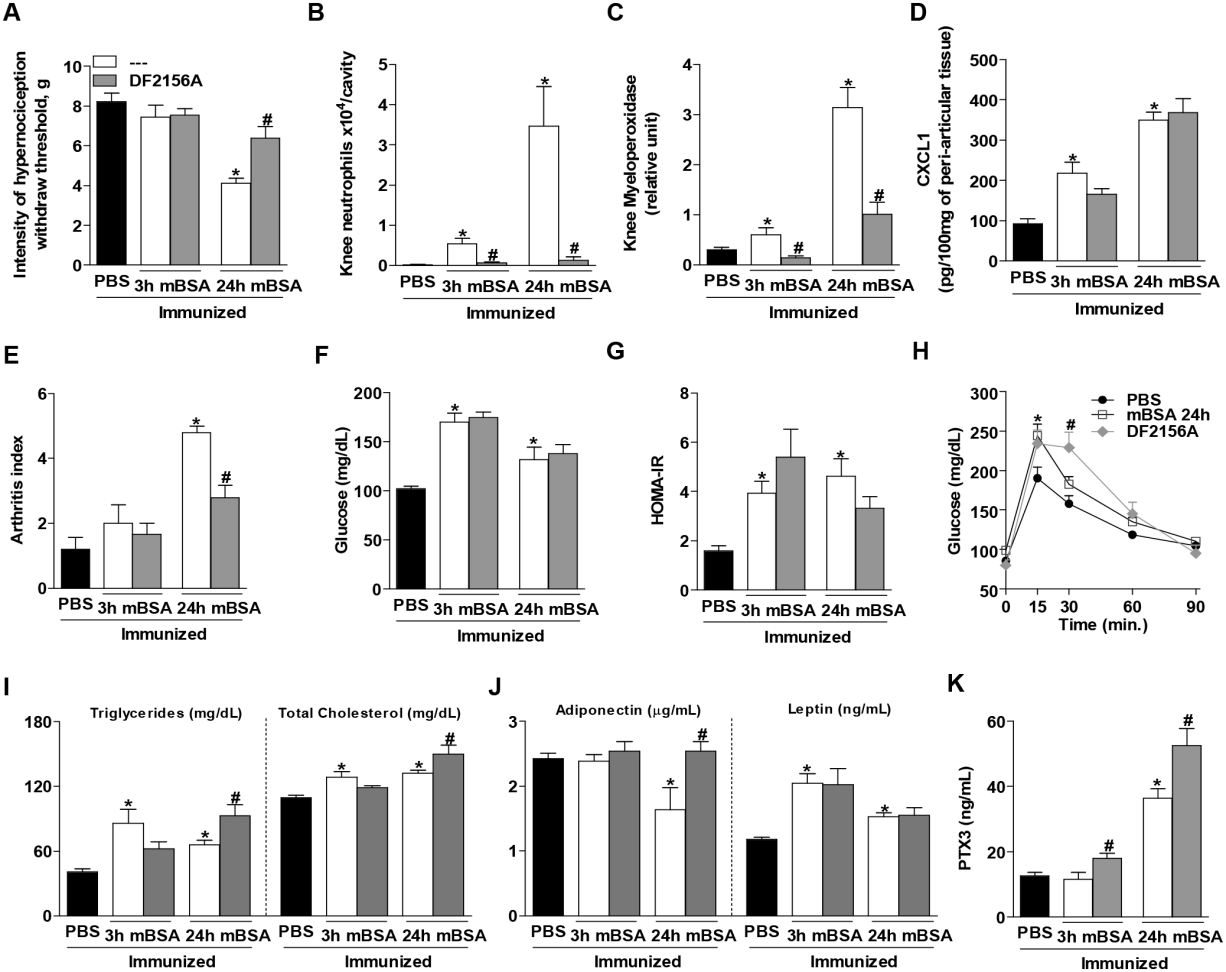
Pre-treatment with DF2156A contributes to metabolic alterations in antigen-induced arthritis mice. (A) Intensity of nociception. (B) Number of neutrophils, (C) myeloperoxidase activity, (D) CXCL1 chemokine in the peri-articular tissue and (E) arthritis index. Systemic glucose metabolism showed by (F) glucose levels, (G) HOMA-IR index and (H) oral glucose tolerance test. The lipids, (I) triglyceride and total cholesterol levels. The adipocytokines, (J) adiponectin and leptin levels. (K) PTX3 levels of mice that received a knee intra-articular injection of PBS, mBSA or mBSA treated prior with DF2156A. Bars represent the mean values±SEM (n = 6–8), *P<0.05 vs. PBS; #*P*<0.05 vs. AIA of the respective time.

The inflammatory response developed in this model of arthritis is characterized by marked neutrophil influx, neutrophil-dependent joint damage and dysfunction [9]. Next, we investigated whether neutrophils, which were reduced by Etanercept, could contribute to the metabolic changes observed in AIA mice. Mice pre-treated with DF2156A, a CXCR1/2 receptor antagonist, showed reduced intensity of nociception, neutrophil accumulation into the knee joint and in the peri-articular tissue following AIA challenge (Fig 4A–4C), although no alterations in the CXCL1 levels were found (Fig 4D). Joint damage, as assessed histologically was also improved in DF2156A-treated mice (Fig 4E). However, the treatment with DF2156A did not alter changes in glucose levels and HOMA-IR induced by AIA (Fig 4F and 4G), and actually, the glucose intolerance worsened (Fig 4H). Similarly, treatment with DF2156A tended to enhance further the levels of triglycerides and total cholesterol (24 hours) (Fig 4I). Levels of adiponectin were further enhanced at 24 hours by the treatment with DF2156A whereas levels of leptin were similarly increased in DF2156A and vehicle-treated animals (Fig 4J). It was observed increased PTX3 levels in AIA mice, especially 24 hours after challenge. The treatment with DF2156A induced a further increase in PTX3 levels at both time points evaluated (Fig 4K).

**Fig 5:**
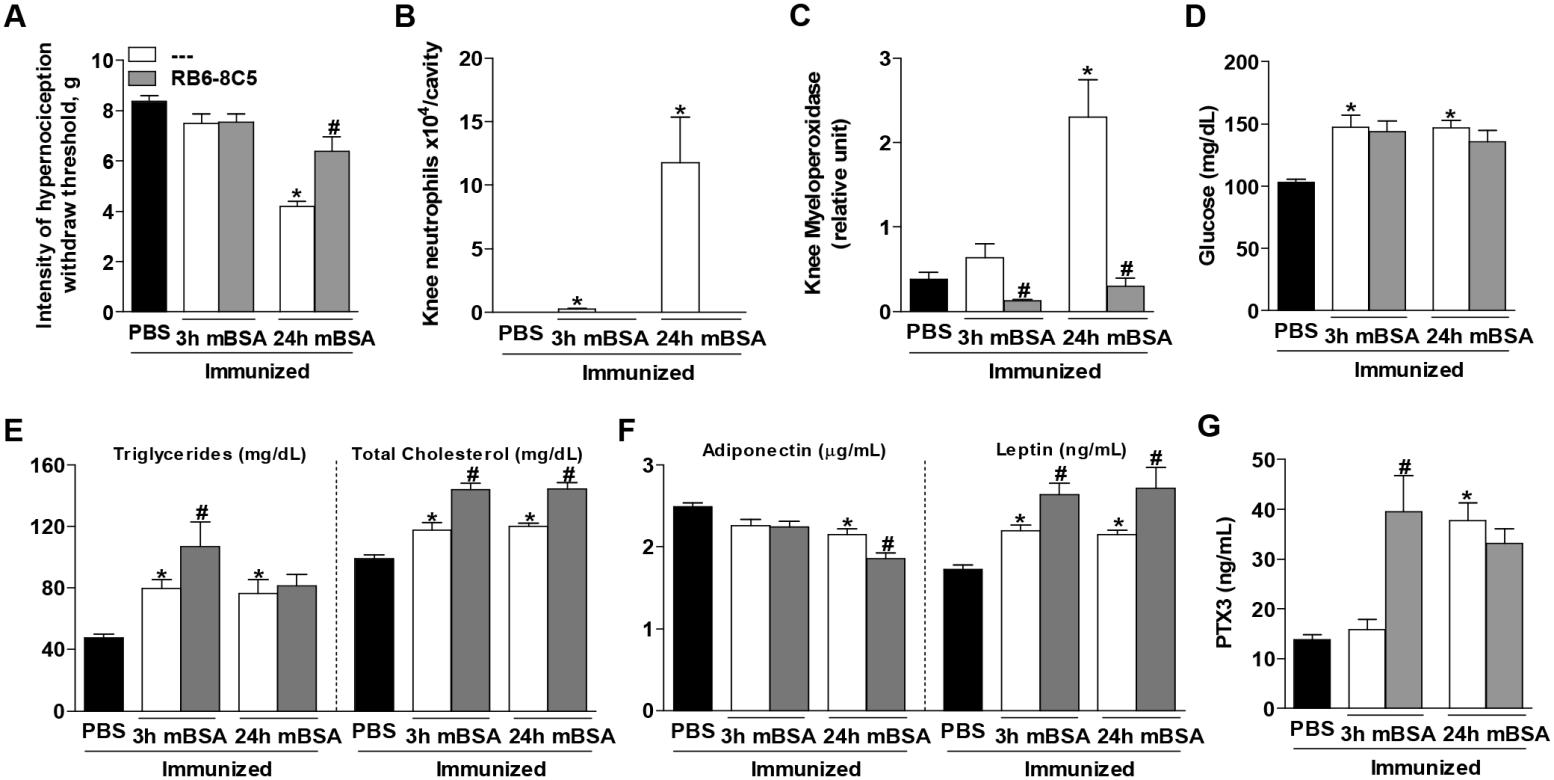
Depletion of neutrophils did not improve the metabolic alterations in antigen-induced arthritis mice. (A) Intensity of nociception. Inflammation in the knee cavity represented by (B) number of neutrophils and (C) myeloperoxidase activity. Metabolic features represented by (D) glucose, (E) triglyceride and total cholesterol levels, accompanied by the adipocytokines (F) adiponectin and leptin levels in the serum. (G) The PTX3 of mice that received an intra-articular injection in the knee cavity of PBS, mBSA or mBSA treated prior with RB6-8C5. The bars represent the mean values±SEM (n = 4–6), *P<0.05 vs. PBS; #*P*<0.05 vs. AIA of the respective time.

In other to better investigate the role of neutrophils in the systemic metabolic alterations observed in AIA mice, we used the monoclonal antibody RB6-8C5 (anti-GR-1) to deplete neutrophils. We confirmed the depletion of neutrophils in these mice by flow cytometry and differential leukocyte count in the blood (S1 Fig), with no alteration on the absolute amount of lymphocytes or monocytes (data not shown). The depletion was also This treatment was effective in reducing hypernociception (Fig 5A). Corroborating with the efficacy of neutrophils depletion, we did not observe increased neutrophil accumulation into the knee joint or in the peri-articular tissue in RB6-8C5-treated mice after AIA induction (Fig 5B and 5C). Analyses of systemic metabolism showed a similar profile to that seen in DF2156A-treated mice. Again, the neutrophil depletion did not prevent the metabolic dysfunction induced by arthritis. There was no alteration on glucose levels in neutrophil-depleted mice compared with AIA groups (Fig 5D). Moreover, triglyceride and cholesterol levels increased further in neutrophil-depleted mice 3 hours after challenge (Fig 5E). Levels of adiponectin reduced at 24 hours and leptin increased further (Fig 5F) in neutrophil-depleted mice compared with AIA control mice. As observed in DF2156A-treated mice, levels of PTX3 tended to be higher after neutrophil depletion than in control AIA mice (Fig 5G). These observations suggest that the metabolic dysfunction in this model of AIA is not associated with the influx of neutrophils. On the contrary, blockade of neutrophil influx or depletion of neutrophils was associated with a mild worsening of systemic metabolic parameters.

## Discussion

Arthritis is frequently associated with metabolic dysfunction, which may be a consequence of the systemic inflammatory response of this disease [5, 19]. Herein, we have shown that the acute model of AIA transiently alters the metabolism of mice, as observed by higher glucose and lipid levels, altered adipocytokine production and by the increase in neutrophil infiltration into the adipose tissue and liver. TNF appears to be an important mediator of the metabolic changes in AIA mice. The investigation of the mechanisms that lead to metabolic alterations ruled out an important participation of the neutrophils, the main cell type involved in the inflammatory response in this model.

The metabolic syndrome is a risk factor for patients with rheumatoid arthritis (RA) [1] and the cause of this dysfunction is still unclear. Some studies have demonstrated that RA patients present alterations in the glucose and lipid metabolism, including insulin resistance, high levels of cholesterol and hypertriglyceridemia in the absence of obesity [20, 21]. Indeed, we have shown that transient metabolic changes may occur even in lean mice during acute arthritis induction. The inflammation is known to induce an increase in the metabolic demand [22]. This increase appears to be transient, but necessary to provide the appropriate fuel for an acute inflammatory response and subsequent recover to homeostasis. The inflammation observed in the adipose tissue and liver may also trigger the mobilization and systemic release of nutrients, as demonstrated in other acute inflammatory processes [23, 24]. It is also described that the intermediated metabolism (gluconeogenesis and lipolysis) is activated during the process of stress [25], leading to increase in lipids and glucose in the circulation [26, 27]. Moreover, the alteration of these parameters may be a consequence of systemic inflammation [28] and be involved with pain, which is often observed in patients with arthritis [29]. Acute pain by itself was associated to insulin resistance, increasing serum levels of glucose and free fatty acids in humans [30]. However, there is a lack of studies showing the association between pain and metabolic alterations apart of the context of inflammation.

TNF is a major cytokine in the context of experimental and human arthritis. We showed that anti-TNF therapy (by using Etanercept) successfully reduced the acute metabolic changes after AIA in mice. This is consistent with studies showing that anti-TNF therapy improves the disease and ameliorates the insulin sensibility and lipid levels in RA patients [7, 31]. In rabbits, activated fibroblast-like synoviocytes are important sources of TNF in antigen-induced arthritis model [32], which could also be the main source of TNF in our model. TNF is a cytokine involved in the expression of many other cytokines that act in the host defense, and contributes to the production of acute phase proteins [33, 34]. The PTX3, an acute phase protein, is produced by leukocytes [18] and its synthesis is induced by cytokines such as TNF and IL-1β [35, 36]. This axis appears to be important to trigger the metabolic changes observed in our study. In fact, mice treated with anti-TNF showed low levels of PTX3 along with some improvement on metabolic changes. In addition to contribute to acute inflammation, there are much data demonstrating that TNF also participates in the resolution of inflammation and tissue remodeling [37]. These latter effects of TNF may be relevant in chronic RA patients as tissue remodeling may help adapting to the chronic inflammatory stimulation and chronic metabolic stress.

TNF is also synthesized in the adipose tissue and may act on glucose metabolism through the increase in lipolysis [38], and consequently contribute to insulin resistance [39]. This cytokine may also alter the production of adipocytokines, which are involved in the inflammatory process as well as metabolism [40]. Adiponectin is considered an anti-inflammatory cytokine and is associated to the insulin sensitivity [41]. Controversially, patients with RA showed an increase in adiponectin levels that are associated to the disease progression [42]. The decrease in this cytokine observed in our model may be due to the acute inflammation induced by the arthritis, yielding the insulin insensitivity. Leptin, which is a pro-inflammatory adipocytokine, is also increased in RA patients as a predictor of disease duration and development [43]. Although leptin may contribute to the systemic inflammation and consequently metabolic alterations, the higher levels of this cytokine following AIA may be also a consequence of the acute inflammatory response. Indeed, recent findings showed that leptin secretion from the adipose tissue is induced few hours after LPS i.p. injection [37]. Combined, these data suggest that metabolic changes appear to be linked to the acute increase of pro-inflammatory mediators rather than neutrophils. Moreover, our data suggest that TNF is a major driver of adipocytokines in the context of acute inflammation. Therefore, it appears that inflammatory mediators are the primary factor driving systemic metabolic changes in arthritis.

Neutrophils and cytokines have a critical role in the inflammatory response observed in RA and in different models of arthritis [44, 45]. A possible explanation for the accumulation of neutrophils in adipose tissue and liver following the challenge into the knee could be related to soluble mediators released by joint resident cells, such as TNF, although we could not detected it in serum. Moreover, the previously migrated neutrophils into the joint could reach the circulation again [46], a phenomenon called reverse transmigration, which, in turn, could accumulate in other organs, as observed in adipose tissue and liver. In the context of RA, the role of neutrophils is more controversial since these cells are not majority in patients with severe disease. However, there is evidence that neutrophil influx does occur during recurrence of disease and may contribute to joint damage [4]. It is known that neutrophils contribute to the production of cytokines [47], reactive oxygen species [48] in an acute inflammatory response. Neutrophil may also contribute to metabolic changes including insulin resistance and liver steatosis, induced by obesity [49, 50]. In our experiments, the use of a CXCR1/2 antagonist did not improve the systemic metabolic alterations observed in AIA mice. However, we did have concerns whether these results were due to the presence of some neutrophils still activated in the circulation. Thus, we depleted neutrophils. Again, the acute systemic metabolic alterations of AIA mice were still present, even in the absence of neutrophils. Moreover, the levels of PTX3 were even increased after the inhibition of neutrophil recruitment or their depletion. Therefore, the worsening of some metabolic parameters in AIA mice may be associated to the increase in systemic inflammatory response that appears to be involved in the production of cytokines.

In summary, our data showed that the induction of local joint inflammation in mice provoked acute alterations in systemic metabolic parameters that were accompanied by an influx of neutrophils into the adipose tissue and liver. The cytokine TNF appears to significantly contribute to the acute systemic metabolic changes. However, the influx of neutrophils does not account for the metabolic changes observed after AIA. We suggest that the inflammatory cytokines may be the main factors involved in the improvement of the altered systemic metabolic parameters induced by anti-TNF therapy.

## Supporting Information

S1 Fig The depletion of neutrophils in the blood of mice treated with RB6-8C5 MAb clone. Analysis of blood cells in the mice that received an intra-articular injection in the knee cavity of PBS, mBSA or mBSA treated prior with RB6-8C5. It is represented the absolute number of (A) total leukocytes and (B) neutrophils. (C) Frequency of total neutrophils (GR1^+high^ CD11b^+high^) by flow cytometry. The bars represent the mean values±SEM (n = 4–6). (D) Representative dot plots of neutrophils as frequency of parent (GR1^+high^ CD11b^+high^). *P<0.05 vs. PBS; #*P*<0.05 vs. AIA of the respective time.
(TIF)
