# Supplementary figures and images for: Tumor Necrosis Factor, but Not Neutrophils, Alters the Metabolic Profile in Acute Experimental Arthritis
Source: PLoS One. 2016 Jan 7;11(1):e0146403. doi: 10.1371/journal.pone.0146403 (PMC4712146; doi:10.1371/journal.pone.0146403)

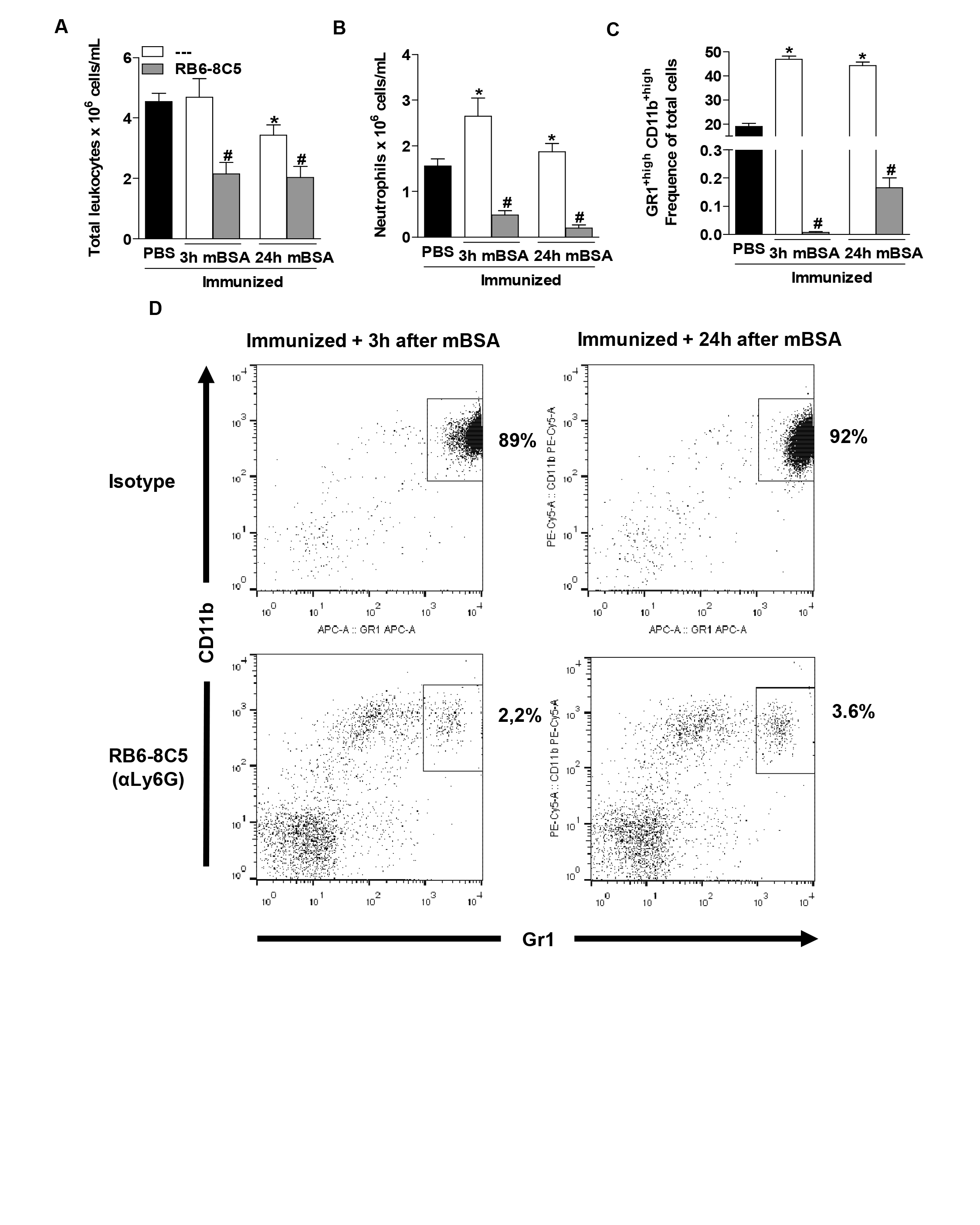

Supplement: S1 Fig — Analysis of blood cells in the mice that received an intra-articular injection in the knee cavity of PBS, mBSA or mBSA treated prior with RB6-8C5. It is represented the absolute number of (A) total leukocytes and (B) neutrophils. (C) Frequency of total neutrophils (GR1+high CD11b+high) by flow cytometry. The bars represent the mean values±SEM (n = 4–6). (D) Representative dot plots of neutrophils as frequency of parent (GR1+high CD11b+high). *P<0.05 vs. PBS; #P<0.05 vs. AIA of the respective time. (TIF) [file pone.0146403.s001.tif]
